# Multimodal Imaging of Immune Checkpoint Inhibitor Myocarditis

**DOI:** 10.3390/jcm14082850

**Published:** 2025-04-21

**Authors:** Shreyans Patel, Kartikeya Dave, Mario J. Garcia, Carlos A. Gongora, Mark I. Travin, Lili Zhang

**Affiliations:** 1 Division of Cardiology, Department of Medicine, Montefiore Medical Center, Albert Einstein College of Medicine, Bronx, New York, NY 10461, USA; mariogar@montefiore.org (M.J.G.); cgongora1@montefiore.org (C.A.G.); lilizhan@montefiore.org (L.Z.); 2 Department of Medicine, Montefiore Medical Center, Albert Einstein College of Medicine, Bronx, New York, NY 10461, USA; kdave@montefiore.org; 3 Division of Nuclear Medicine, Department of Radiology, Montefiore Medical Center and the Albert Einstein College of Medicine, Bronx, New York, NY 10461, USA; mtravin@montefiore.org

**Keywords:** cardiovascular magnetic resonance imaging, echocardiography, immune checkpoint inhibitor, myocarditis, positron emission tomography

## Abstract

Immune checkpoint inhibitors (ICIs) have dramatically changed the landscape of cancer treatment and are increasingly used either as monotherapy or in combination with other ICIs, chemotherapy, and molecularly targeted agents. ICI myocarditis is a rare but potentially fatal irAE associated with the use of ICI characterized by T-cell mediated cardiomyocyte death. Diagnosing ICI myocarditis can be intricate as its atypical presentations. Multimodal imaging plays a crucial role in the diagnosis and risk stratification of ICI myocarditis. Current management strategies for ICI myocarditis include corticosteroids and immunosuppressants. Multidisciplinary collaboration is vital in these cases—combining oncology expertise with cardiology insights.

## 1. Introduction

Immune checkpoint inhibitors (ICIs) are a class of medications (monoclonal antibodies) that block the binding of immune checkpoint proteins (e.g., CTLA-4, PD-1, and LAG-3) on T-cells with their partner proteins (e.g., PD-L1) on cancer or antigen-presenting cells, preventing a downregulation in the activity of T-cells and increasing their cancer killing activity [1]. The US FDA has approved four different categories of ICIs, namely, PD-1 inhibitors, PDL-1 inhibitors, CTLA-4 inhibitors, and LAG -3 inhibitor. ICIs have dramatically changed the landscape of cancer treatment and are used either as monotherapy or in combination with other ICIs, chemotherapy, and molecularly targeted agents. Between 2015 and 2023, eleven ICI drugs were approved for 88 different indications for 20 general tumor types. The estimated eligibility for ICIs has increased from 1.54% in 2011 to 55.47% in 2023 [2].

As ICIs remove one of the safeguards that prevent overactivity of the immune system, they may lead to a new spectrum of immune-related adverse events (irAEs). ICI myocarditis is a rare but potentially fatal irAE associated with the use of ICIs that is characterized by T-cell mediated cardiomyocyte death. There is a wide range in the reported incidence of ICI-associated myocarditis, likely due to the difficulty in establishing the diagnosis and the lack of any standardized assessment protocols (Table 1). The reported incidence is generally low at 0.27–1% [3]. However, the rate of major cardiovascular outcomes can be as high as 40% in patients diagnosed with ICI myocarditis [4,5,6]. The median time of onset from the initiation of ICI noted in various studies is 34 to 167 days [4,7,8].

### 1.1. Risk Factors

Limited studies have examined the risk factors for ICI myocarditis. Consistent data suggest that patients receiving combination ICI therapies have an increased incidence of myocarditis and increased mortality compared to those receiving monotherapy [4,5,8]. Zamami et al. noted that females had a 1.92 times higher likelihood of developing ICI myocarditis [15]. A BMI > 27.8 kg/m^2^ may be associated with a higher risk of ICI myocarditis [4,16,17]. Some studies have suggested that an older age (>75 yrs) was associated with a risk of developing ICI myocarditis [6,15,16,17], while others did not find an association [16,18,19]. There is conflicting evidence on the associations between cardiovascular risk factors and ICI myocarditis. Bragheiri et al. showed that apart from hypertension, traditional cardiovascular risk factors and comorbidities, such as diabetes mellitus, hyperlipidemia, coronary artery disease, atrial fibrillation, cerebrovascular accidents, and heart failure, were not associated with ICI-rM [7]. Conversely, some retrospective studies have noted a possible association of ICI myocarditis with use of cardiovascular medications prior to starting ICI therapy, indicating a possible association between cardiovascular risk factors and ICI myocarditis [16]. An elevated baseline absolute neutrophil count may be associated with an increased risk of developing ICI myocarditis [7].

### 1.2. Pathogenesis

Biopsy specimens from patients with ICI myocarditis showed a patchy lymphocytic infiltrate of CD4+ and predominantly CD8+ T cells [9,20]. This was also seen in mouse models such as PD1-deficient mice on Balb/c background, A/J mice treated with anti-PD-1 antibodies, and Ctla4^+/−^Pdcd1^−/−^ mice. The cardiac-specific protein (α-myosin heavy chain)-reactive CD8+ T cells that have high expression of granzyme and IFN-γ seem to be the main drivers of ICI myocarditis in mouse models [21,22,23]. It is postulated that the persistence of these cardiac myosin-specific T cells in the peripheral tissues and their subsequent activation due to checkpoint blockade result in myocarditis [21].

### 1.3. Clinical Features

The clinical presentation of ICI myocarditis can be variable, ranging from mild symptoms, including fatigue, dyspnea, orthopnea, chest pain, palpitation, lower extremity edema, myalgia, and lightheadedness, to severe fulminant cases presenting with cardiogenic shock, complete heart block, intractable ventricular arrhythmias, or cardiac arrest [19,24]. It may also be incidentally discovered as an asymptomatic troponin elevation and confirmed on CMR or EMB, and is frequently associated with other irAEs like myositis and myasthenia gravis [4,8,25,26].

### 1.4. Biomarkers

Frequently elevated biomarkers in ICI myocarditis patients include CK, troponin-I (cTnI) and troponin-T (cTnT). While both cTnI and cTnT may be used for the diagnosis of acute myocardial infarction (a condition that may need to be differentiated from ICI myocarditis), some studies have indicated that cTnI may be more specific than cTnT, which may be elevated in patients with immune-related myositis [27,28,29]. However, cTnT was suggested to be more sensitive, as Lehman et al. [30] found that increased rate of positivity for cTnT (93%) versus cTnI (64%) in 60 patients with ICI myocarditis and a cTnT:URL ratio <32 within 72 h of admission were associated with a subgroup at low risk of MACEs (major cardiac adverse events). Additionally, a cTnI elevation was noted to have a rapid rise and decline compared to a more prolonged and sustained cTnT elevation, suggesting that a normal value of cTnI at the time of admission should prompt a concomitant cTnT measurement. ICI myocarditis was also associated with elevations in AST, ALT, and CPK levels. Among the noncardiac biomarkers, only CPK was associated (per 100% increase) with the development of myocarditis (HR: 1.83; 95% CI: 1.59–2.10) and all-cause mortality (HR: 1.10; 95% CI: 1.01–1.20) in a multivariable analysis. This study suggested that increases in the levels of noncardiac biomarkers during ICI treatment, notably CPK, should prompt a further evaluation of ICI myocarditis [10].

### 1.5. Electrocardiography

Mahmood et al. noted that while pretreatment ECGs were normal in the majority of patients, ECGs after ICI myocarditis was diagnosed were almost always abnormal (89%) [4]. A normal ECG does not rule out ICI myocarditis. Common abnormalities include sinus tachycardia, repolarization abnormalities, conduction defects, and atrial and ventricular arrhythmias [9,31]. An increased QRS duration is frequently seen in patients with ICI myocarditis and may predict an increased risk of MACEs (cardiovascular death, cardiogenic shock, cardiac arrest, and hemodynamically significant heart block) [17]. Patients with complete heart block and life threatening ventricular arrhythmia are more likely to experience all-cause or cardiovascular mortality [11,31].

## 2. Multimodal Imaging

**Figure 1:**
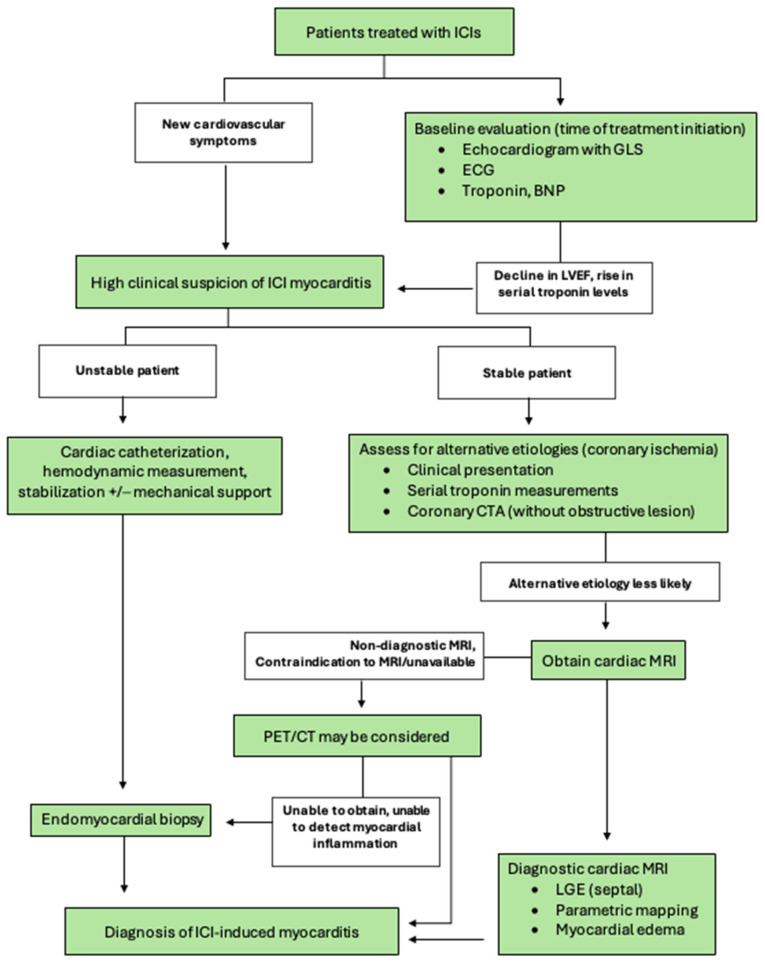
Diagnostic algorithm for ICI myocarditis. Proposed algorithm for the diagnosis of ICI myocarditis. Changes from the baseline evaluation and the development of new cardiovascular symptoms should prompt further evaluation. Unstable patients should be triaged to a more invasive assessment and stabilization with eventual endomyocardial biopsy (EMB). Stable patients should undergo cardiac MRI (CMR), which if nondiagnostic can consider EMB if the symptoms are persistent. ICI = immune checkpoint inhibitor. ECG = electrocardiogram. BNP = brain natriuretic peptide. PET/CT = positron emission tomography/computerized tomography. LGE = late gadolinium enhancement.

The International Cardio-Oncology Society and European Society of Cardiology published similar guidelines for the diagnosis and evaluation of ICI myocarditis, highlighting the essential role of multimodal imaging (Table 2). These guidelines focus on ruling out alternative diagnoses such as acute coronary syndrome and infection before using biomarkers (troponin level) and imaging modalities to confirm the diagnosis of myocarditis. It is noted that the current level of evidence is mostly from small retrospective studies, case series, or expert opinions. Below, we present current evidence concerning the prominent imaging modalities and propose a stepwise algorithm for the diagnosis of ICI myocarditis (Figure 1).

### 2.1. Echocardiography

Structural and functional evaluations by echocardiography are the first-line tool in the diagnosis of ICI myocarditis due to its comprehensive assessment, wide availability, and ease of performance. Serial assessments of LVEF are an advantage of echocardiography but these can be limited by interobserver variability, the operator’s experience, geometric assumptions for its calculation, the loading state of patients, echocardiographic windows, and image quality. In addition, echocardiography has shown poor sensitivity in the detection of early subclinical myocardial dysfunction [33,34]. LV dysfunction is not a key diagnostic criteria for ICI myocarditis because more than half of the ICI myocarditis patients have preserved systolic function [4,8]. Myocardial strain by speckle tracking echocardiography is an established tool for the diagnosis of cardiotoxicity due to its ability to detect subclinical dysfunction before a decrease in LVEF [35]. There remains a paucity of guidelines concerning the use of myocardial strain in the diagnosis of ICI myocarditis, although some studies have highlighted its utility. Awadella et al. demonstrated that global longitudinal strain (GLS) was decreased in patients with ICI myocarditis compared to a control group with preserved or reduced LVEF [35]. Each percent decrease in GLS was associated with 1.5 percent relative increase in MACEs in patients with a reduced LVEF and an even greater 4.4 percent relative increase in those patients with a preserved LVEF. A study by Tamura et al. demonstrated that an early relative decrease in basal longitudinal strain (>10%) was associated with an increase in the troponin level and development of myocarditis in patients on ICIs [36]. The global circumferential strain (GCS) and global radial strain (GRS) were also lower in patients with myocarditis compared with on-ICI controls. GCS (HR: 4.9 [95% CI: 1.6–15.0]; *p* = 0.005) and GRS (HR: 3.9 [95% CI: 1.4–10.8]; *p* = 0.008) below the medians were associated with an increased event rate. Additionally, the significant incremental prognostic utility of GRS over LVEF and GCS over cTnT was demonstrated [37]. Despite the widespread variation in vendors and training, growing evidence is corroborating the utility of myocardial strain in the diagnosis and prognostication of ICI myocarditis.

Echocardiography can reliably detect the presence of pericardial effusion, which has been documented in 7–17% of ICI myocarditis patients, but there remains a lack of evidence on how an echocardiographic assessment of pericardial disease, valves, and diastolic function plays a role in the diagnosis and risk stratification of ICI myocarditis. Current guidelines recommended assessing ECG, BNP, and cTn measurements in all patients before starting ICI therapy, and baseline echocardiography is recommended in high-risk patients before starting ICI therapy [38,39].

### 2.2. Cardiac Magnetic Resonance Imaging

Cardiac magnetic resonance imaging (CMR) is considered the gold standard non-invasive test in the diagnosis and risk stratification of ICI myocarditis [40]. CMR is advantageous in tissue characterization and has a greater reproducibility in assessing LVEF using serial imaging compared to echocardiography.

The diagnosis of myocarditis relies on the modified Lake Louise criteria, which include the presence of non-ischemic myocardial injury and myocardial edema as the main criteria. Based on the current data, however, late gadolinium enhancement (LGE) may be present to a lesser degree in patients with ICI myocarditis than in those with non-ICI mediated myocarditis and on its own may not be able to exclude ICI myocarditis. Zhang et al. demonstrated that only 48% of ICI myocarditis patients had LGE and only 28% had elevated T2-weighted STIR (short-T1 inversion recovery) in a cohort of 103 patients [41]. However, LGE has been shown to be typically present in 80% of non-ICI myocarditis patients [42,43]. Therefore, only relying on LGE or T2-weighted STIR to diagnose ICI myocarditis is inadequate. A study comparing ICI myocarditis to viral myocarditis by Cadour et al. highlighted that LGE was less frequent in patients with ICI myocarditis than those with viral myocarditis, but was more likely to involve the septal segments and midwall layer [44]. Septal LGE was the only CMR predictor of MACE at 1 year, even after adjustment for peak troponin levels [44]. Another factor in LGE detection is time point at which the CMR was obtained; as demonstrated by Zhang et al., LGE detection increased from 21.6% to 72.0% when MRI was obtained on day 4 of symptoms and onward [41].

Applying the modified Lake Louise Criteria, including T2-weighted images and LGE along with T1 and T2 mapping and ECV, may offer improved diagnostic sensitivity. In 136 patients diagnosed with ICI myocarditis and with a CMR, 95% of ICI myocarditis patients met the non-ischemic myocardial injury criteria and 53% met the myocardial edema criteria by adding T1 and T2 mapping. Native T1 values had excellent discriminatory value for subsequent MACEs, with an area under the curve of 0.9 and T1 mapping was more predictive of MACEs as compared to T2 mapping [45]. In a retrospective study of 56 patients with ICI myocarditis by Zhao et al., an elevated T2 value was present in 92% of patients in contrast to a native T1 value (73%), myocardial edema (52%), and LGE (63%) [46]. Additionally, a case series of four patients with suspected ICI myocarditis revealed substantially increased T1 values, while T2 values were only mildly increased or normal. CMR demonstrated an improvement in the findings after immunosuppressive treatment in two patients with follow-up imaging. There was only limited agreement between the degree of high-sensitive troponin levels and T1/T2 levels, suggesting that CMR may be an alternative strategy to monitor the treatment response and to decide the timing of tapering/stopping immunosuppression [47]. In another study including 22 participants undergoing ICI therapy and with a CMR before and after receiving ICIs, compared with the baseline CMR, participants displayed increased markers of diffuse myocardial edema (T1 relaxation time, T2 relaxation time, and T2 signal intensity ratio) and decreased left ventricular average systolic longitudinal strain at the follow-up CMR. New nonischemic LGE lesions were prevalent in 9% participants and small pericardial effusions were more evident at follow-up in 45% participants. However, ICI-related myocarditis was not clinically suspected in any participant at the time of follow-up, although 27% participants had mild to moderate noncardiac irAEs [48]. These results suggest that subclinical myocarditis can present in patients after ICI therapy, and whether subclinical myocardial inflammation can lead to long-term cardiac dysfunction is unclear.

Overall, the body of evidence for the clinical utility of CMR in the diagnosis of ICI myocarditis is growing. Though the current evidence is largely gathered from small cohort studies due to the low prevalence of ICI myocarditis (0.27–1%), the available data support CMR as a valuable diagnostic tool in this population.

### 2.3. Computed Tomography

The traditional use of Computed Tomography (CT) imaging in oncologic management has been focused on mitigating the atherosclerotic cardiovascular risk in patients undergoing cancer treatment [49]. At present, data on the role of CT in monitoring and surveillance for ICI myocarditis are scarce. Coronary CT angiography (CCTA) can be a useful tool in identifying an etiology for a reduction in LVEF or rise in cardiac biomarkers given its high sensitivity for ruling out obstructive coronary artery disease [50]. However, the accuracy in assessing the severity of calcified plaques can be limited by significant coronary calcification, which may be more present in an elderly population, especially those who have undergone chest radiation therapy. Additionally, increasing evidence showed that ICIs may increase the risk of atherosclerotic cardiovascular events by potentially accelerating atherosclerosis, and CCTA can be instrumental to examine the coronary plaque burden and progression [50,51]. CT in combination with Positron emission tomography (PET) may become a valuable diagnostic tool for ICI myocarditis, as will be discussed below.

### 2.4. Nuclear Imaging

A multimodal approach for the evaluation of ICI myocarditis is becoming integral, and nuclear imaging is emerging as an important component. Traditionally, Single-Photon Emission Computed Tomography (SPECT) and Multigated Acquisition Scan (MUGA) imaging are readily available and can provide utility in ruling out coronary ischemia and obtaining reproducible and accurate measurements of LVEF in cardio-oncology practice. PET/CT can detect myocardial inflammation and has the potential to be a valuable modality for detection of ICI myocarditis [52,53]. Fludeoxyglucose (FDG) has been the primary tracer investigated thus far in myocarditis; however, there are currently limited and inconsistent data on the use of FDG-PET in ICI myocarditis patients. In patients with suspected myocarditis analyzed using integrated PET/MRI, compared with CMR (LGE and/or T2), the sensitivity and specificity of PET was 74% and 97%. The overall spatial agreement between PET and CMR was κ = 0.73. The spatial agreement between PET and T2 (κ = 0.75) was higher than the agreement between PET and LGE (κ = 0.64) [54]. Ederhy et al. demonstrated that abnormal cardiac fixation on FDG-PET (suggestive of myocarditis) was observed in only 2/21 patients with otherwise definite ICI-myocarditis versus 1/7 patients without ICI myocarditis, indicating very limited utility in the diagnosis of ICI myocarditis [55]. A more recent study by Tong et al. has demonstrated that in a small cohort of patients (*n* = 12), there was agreement between CMR and PET in seven cases and discordance in five cases, suggesting the complimentary role of hybrid PET-CMR for the timely diagnosis of ICI myocarditis [56].

Novel nuclear tracers are being investigated in this population. One such is Ga-DOTA(0)-Phe(1)-Tyr(3)-octreotide (Ga-DOTATOC) PET/CT, which utilizes a somatostatin analog to detect inflammation. This modality may have utility in the diagnosis of ICI myocarditis as it may be able to detect early inflammation that may not be picked up by CMR. In a study by Boughdad et al., all nine (100%) patients imaged with ^68^Ga-DOTATOC PET/CT presented with pathological myocardial uptake in the LV that was suggestive of myocarditis. Of these same patients, eight had CMR, with only three patients (38%) showing evidence of myocarditis [57]. In addition, eight out of nine patients who had undergone Ga-DOTATOC PET/CT were treated with steroids or IVIG prior. These findings suggest that this tracer may have superior sensitivity in the detection of early inflammation suggestive of ICI. In this study, 5/6 patients that also presented with myositis symptoms showed uptake on whole body imaging with Ga-DOTATOC PET/CT, which demonstrates a potential advantage in assessing whole-body inflammation in patients treated with ICIs [57]. Additionally, ^68^Ga-fibroblast activation protein inhibitor (FAPI) PET/CT was studied for the detection of myocardial alterations caused by ICIs in three patients where FAPI PET/CTs showed a cardiac enrichment of the marker in myocarditis patients and less distinct or absent in patients receiving ICIs without any signs of cardiac impairment [58].

Overall, the current data on PET/CT use for ICI myocarditis is limited, but it may be a valuable diagnostic option if CMR is unable to be obtained due to intracardiac devices or patient tolerability. Also, despite the suggested sensitivity for the detection of ICI myocarditis, especially early in its course, PET/CT may be difficult to execute in the acute setting.

### 2.5. Endomyocardial Biopsy

Endomyocardial biopsy (EMB) remains the gold standard for the diagnosis of myocarditis. The Dallas criteria, while not perfect [59,60], are used to determine the presence or absence of myocarditis. Confounding factors include inter-observer variability in the histologic interpretation [60] and sampling error, which can be reduced by increasing sample size to five or more [61]. EMB should be considered for severe cases of suspected ICI myocarditis with inconclusive biomarkers and imaging findings [39]. Johnson et al. noted in their two cases of ICI myocarditis a patchy lymphocytic infiltrate positive for the T cell marker CD3 or macrophage marker CD68. Infiltrating cells were positive for CD4 and CD8 and negative for CD20 and contained no antibody deposits [9]. Sobol et al., in their six cases, noted a lympho-histiocytic infiltrate with numerous CD163-positive and weakly CD4+ histiocytic cells mixed with predominantly CD8+ T cells, many of which were PD-1- and granzyme B-positive [20]. Based on 28 patients who had EMB for suspected ICI myocarditis, Palaskas et al. developed an EMB grading system (grades 0–2) for ICI myocarditis encompassing a spectrum of histologic findings of inflammatory infiltrates and found a subset of patients with low-grade myocardial inflammation who were able to continue ICIs without immunosuppressive therapy [62].

## 3. Diagnosis

A prompt diagnosis of ICI myocarditis is essential to address this potentially fatal complication of immunotherapy, and it should be considered in symptomatic patients who received ICI therapy in the last 12 weeks [63]. In 2019, Bonaca et al. proposed a framework for the diagnosis of ICI myocarditis based on tissue pathology, clinical presentation, imaging, and biomarkers [64]. Recently, the International Cardio-Oncology Society (IC-OS) published a set of criteria to diagnose ICI myocarditis based on clinical, biomarker, imaging, and pathological evidence while also providing a classification of severity during the active and recovery phase of the disease (Table 2) [32]. This set of criteria highlights combining the use of the clinical presentation, biomarkers, multimodal imaging, and EMB to establish the diagnosis of ICI myocarditis. The major and minor criteria from these guidelines serve as the basis for the diagnostic pathway below, as we focus on the use of cardiac biomarkers and imaging to solidify the diagnosis of ICI myocarditis while ruling out alternative etiologies for real-world clinical scenarios.

We propose a diagnostic algorithm focused on the appropriate diagnostic testing based on the symptoms and acuity of the patient’s condition (Figure 1). Initially, a clinical presentation of new cardiovascular symptoms along with elevated cardiac biomarkers and EKG changes in patients treated with ICIs should raise a concern for ICI myocarditis. Depending on the clinical presentations, appropriate differential diagnoses should be considered, such as myocardial ischemia/infarction, pulmonary embolism, sepsis, anemia, or other causes of myocardial injury. The coexistence of other irAEs (i.e., myositis) and longitudinal changes in the clinical presentations and biomarkers can often aid narrowing down to a diagnosis of ICI myocarditis. Echocardiography along with GLS should be utilized as the first line tests to detect any changes in LV systolic function and regional wall motion abnormalities. After ruling out alternative etiologies for the current clinical presentation, the major and minor criteria from the IC-OS criteria (Table 2) are applied to confirm the diagnosis of myocarditis. Unstable patients should be triaged to cardiac catheterization for the invasive measurement of hemodynamics and a definitive diagnosis with endomyocardial biopsy in order to initiate treatment as soon as possible. In stable patients, cardiac MRI should be utilized as the next step in the diagnostic pathway with parametric mapping and an assessment for LGE. In some cases, it may be necessary to rule out myocardial ischemia as a cause of elevated cardiac biomarkers if clinically suspected. Despite limited evidence on PET/CT in this population, it may be considered in selected cases as an adjunct in those patients where cardiac MRI is nondiagnostic or unable to be obtained. We believe this proposed diagnostic pathway allows for the proper balance of establishing a correct diagnosis while also not delaying treatment for the varying acuity of presentations of ICI myocarditis. It has been increasingly recognized that clinically stable patients with low-level myocardial inflammation/injury after ICI treatment can be observed clinically without aggressive immunosuppressive treatment.

### 3.1. Monitoring

Baseline biomarker and ECG testing are recommended for all patients to be started on ICIs. Patients at an elevated risk for ICI myocarditis may be considered to have a baseline echocardiogram [3,39]. Troponin and ECG surveillance before the second, third, and fourth doses of ICIs are recommended only by the ESC 2022 cardio-oncology guidelines as a class IIa indication [39]. Due to the low incidence of ICI myocarditis, the cost effectiveness of this approach remains unclear. Additionally, providers should avoid discontinuing ICIs prematurely if these screening tests become positive because many diagnoses other than ICI myocarditis can incur similar findings, and a careful decision plan should be made by a multidisciplinary team to optimize cancer and cardiovascular outcomes.

Biomarker surveillance with hs-cTnT is currently controversial. Waliany et al. conducted a prospective study of ICI myocarditis surveillance and noted that a high cutoff threshold for hs-cTnT (1000 ng/L, 2000 ng/L) improved the positive predictive value of the test for ICI myocarditis (75%, 100%) as compared to traditional values (≥55 ng/L, 99th percentile value for general population) [65]. P. F. van den Berg et al. investigated the significant rise in hs-TnT levels (100% rise from baseline, with an absolute value ≥2x upper limit of normal (≥28 ng/L)) in 164 patients treated with ICIs and found that 26 patients (16%) exhibited significant hs-TnT elevations, while 8 patients (5%) developed ICI myocarditis. The study concluded that hs-TnT elevations occur more often than previously reported, are often asymptomatic, and do not always lead to a myocarditis diagnosis [66]. Elderly patients and those on combination ICI therapy may have a relatively higher risk of ICI myocarditis, but the overall occurrence remains low; thus, troponin monitoring is generally not recommended in these populations [4,5].

### 3.2. Management of ICI Myocarditis

There are currently no randomized controlled trials (RCTs) specifically evaluating treatment options for ICI myocarditis, and current management relies on expert consensus and case series data [67]. Treatment starts with determination of the illness severity. In all confirmed cases, ICI therapy should be discontinued, and the patient should be hospitalized in a telemetry unit to monitor for arrhythmia and conduction abnormalities. There are various societal guidelines in place detailing approaches to the management of ICI myocarditis (Table 3). In general, initial treatment is with high-dose corticosteroids for at least 3–5 days (i.e., methylprednisolone 500 mg to 1000 mg i.v. daily) and should be started as soon as the diagnosis is strongly suspected or made because the early initiation of corticosteroids is associated with better outcomes [68]. Further management is based on the patient’s clinical status. If the patient is recovering (cTn levels are reduced by >50% peak or arrhythmias are improved), treatment can be switched to a tapering regimen of oral prednisolone (1 mg/kg/day, with 10 mg weekly taper) while monitoring troponin levels [39,69,70]. Therapeutic plasma exchange, plasmapheresis, or intravenous immunoglobulin can be considered in patients with concomitant myasthenia gravis [69,71]. For steroid-refractory ICI myocarditis, second-line immunosuppressants (i.e., abatacept) can be used. Other treatment possibilities include mycophenolate mofetil, rituximab, anti-thymocyte globulin, alemtuzumab, and tocilizumab [39,69,70]. Caution is advised regarding use of infliximab for ICI myocarditis due to its risk associated with heart failure [70]. Patients with smoldering ICI myocarditis are diagnosed incidentally without any clinical signs or symptoms; the optimal treatment course for these patients is unclear but discontinuation of ICIs and steroid taper can be considered [19].

Patients with fulminant ICI myocarditis with hemodynamic instability should be admitted to the intensive care unit and provided with second-line immunosuppression and mechanical circulatory support as needed [1]. Currently, there is a paradigm shift in second-line treatment taking place where intensified initial immunosuppression is combined with corticosteroids or targeted therapies directed at the pathophysiology of ICI myocarditis are used in combination upfront [72,73]. However, the available evidence to support these novel strategies is limited to observational studies and case reports. For example, Salem et al. presented a case in which the use of abatacept (a cytotoxic T-lymphocyte–associated antigen 4 [CTLA-4] agonist) led to the resolution of severe, glucocorticoid-refractory ICI myocarditis [74]. Wang et al. reported that tofacitinib (5 mg twice a day) was used in eleven corticosteroid-resistant patients, with seven patients recovering from ICI myocarditis, showing a promising therapeutic effect [75]. A recently published study by Salem et al. showed that early management of respiratory muscle failure using mechanical ventilation and high-dose abatacept with CD86 receptor occupancy monitoring combined with ruxolitinib may be promising to mitigate the high fatality rates in patients with severe ICI myocarditis [76].

Most patients with ICI myocarditis should not be rechallenged with ICIs after recovery due to the high risk of reoccurrence [1]. However, patients who have preserved cardiac function and have a lower grade of endomyocardial biopsy-proven myocardial injury may be rechallenged on a selective basis with careful monitoring of cardiac biomarkers, though the current evidence is very limited and there are no clear guidelines on this approach [62].

#### Clinical Case

The following case is presented to highlight the importance of multimodal imaging in the diagnosis of ICI myocarditis.

**Figure 2:**
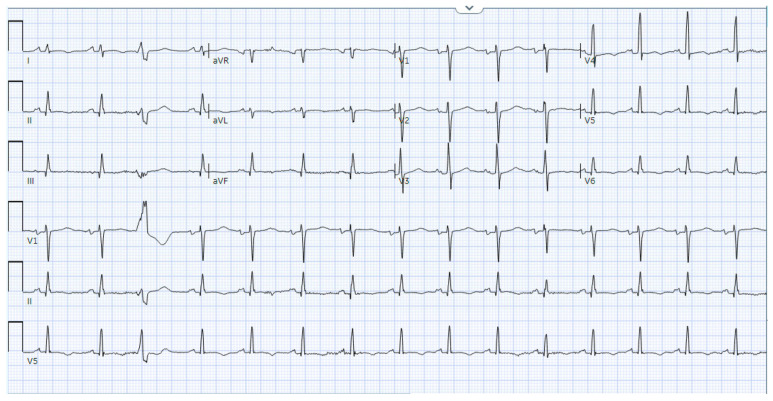
Electrocardiogram from the clinical patient. Electrocardiogram from the clinical patient showing sinus tachycardiac with occasional premature ventricle contractions and nonspecific T wave changes.

**Figure 3:**
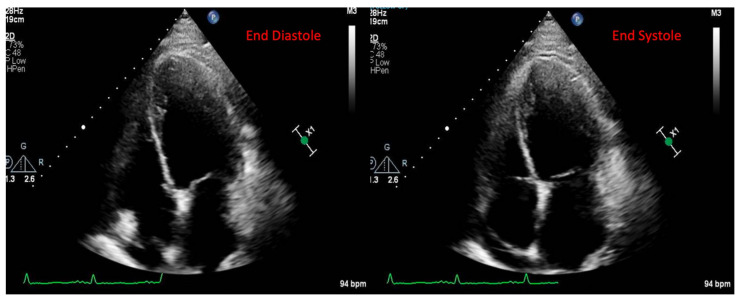
Echocardiogram images from the clinical patient. Transthoracic echocardiogram apical 4-chamber images (shown at end diastole and end systole). Severely decreased left ventricular ejection (30%). Mild right ventricular dysfunction. Small pericardial effusion.

**Figure 4:**
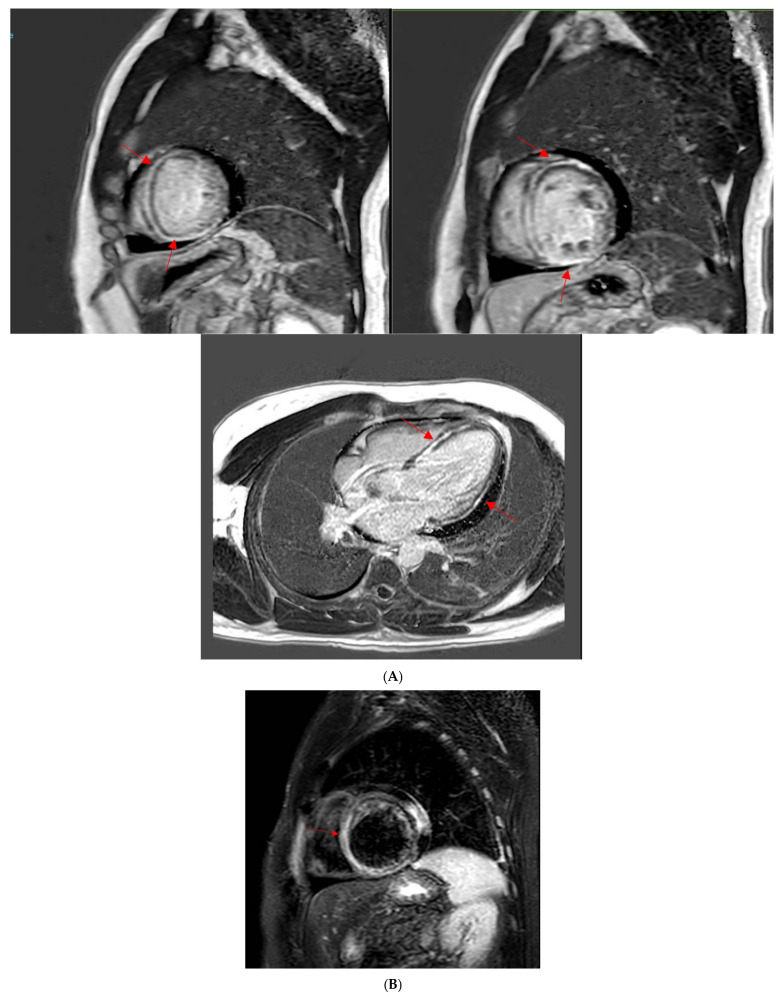

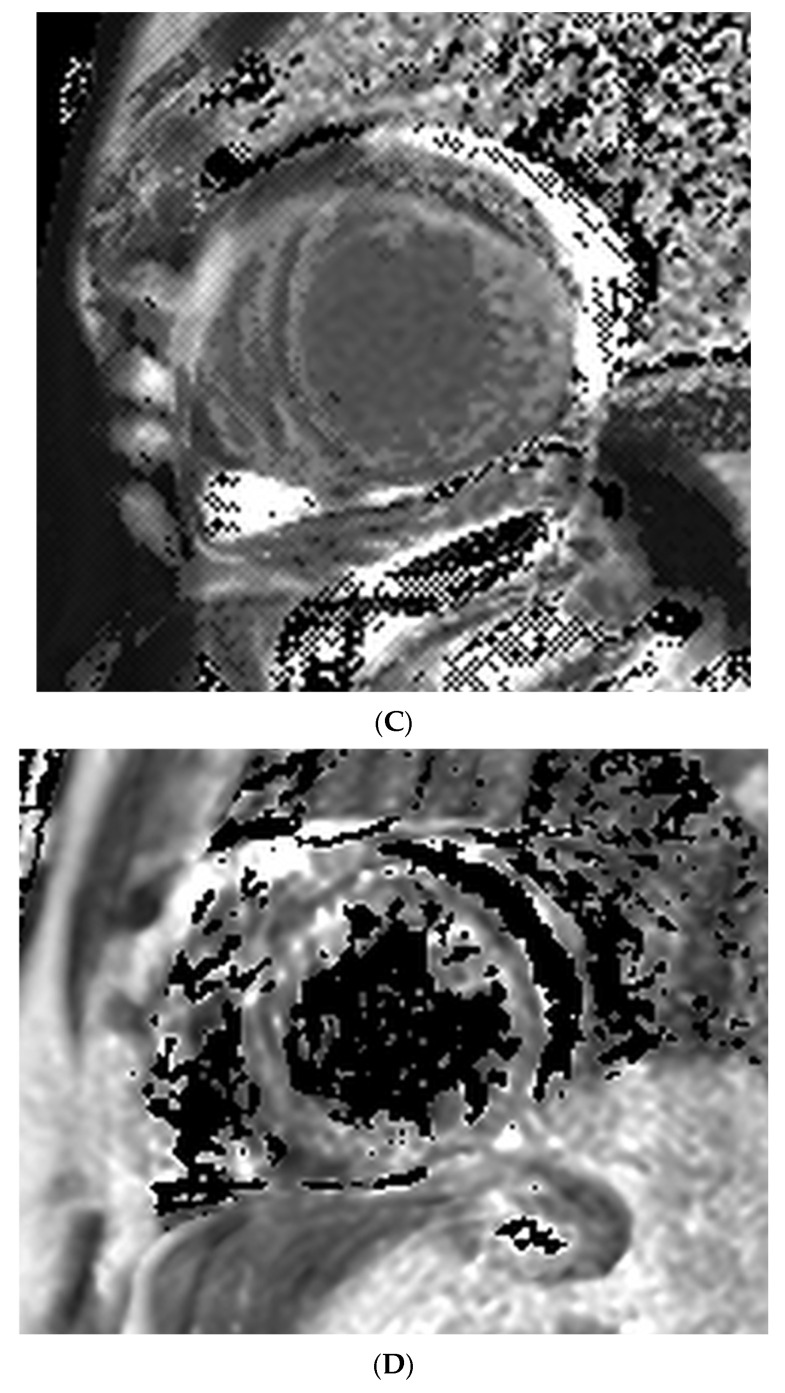
(**A**) Cardiac MRI LGE images from the clinical patient. Cardiac MRI images showing linear midventricular LGE (red arrows) in the basal and mid interventricular septum, extending into the anterior and inferior LV segments. Globally reduced biventricular function (LVEF 29% and RVEF 42%). (**B**) Cardiac MRI T2-weighted images from the clinical patient. Cardiac MRI T2-weighted images. There is interventricular T2 enhancement (red arrow) consistent with edema. (**C**) Cardiac MRI T1 mapping images from the clinical patient. Cardiac MRI T1 mapping images revealed an elevated native T1 map at 1470 ms (normal 1000 +/− 50 ms at 1.5T). (**D**) Cardiac MRI T2 mapping images from the clinical patient. Cardiac MRI T2 mapping images revealed elevated T2 mapping at 70 ms (myocardial edema suggested if T2 > 55–60 ms).

A 49-year-old male patient with a history of human immunodeficiency virus (HIV) was undergoing treatment for Kaposi sarcoma with cabozantinib and nivolumab (ICI) as the third line of treatment. Approximately 2 months after his last treatment, this patient presented to the emergency room with progressive shortness of breath over the last few weeks and was found to be in acute heart failure. An electrocardiogram showed sinus tachycardia with occasional premature ventricular complexes (Figure 2). The troponin I level peaked at 0.62 ng/mL (normal value < 0.03 ng/mL) and the B natriuretic peptide level was 1023 pg/mL (normal value < 100 pg/mL). The baseline echocardiogram prior to treatment initiation showed normal biventricular function, but his echocardiogram at presentation showed severely reduced left ventricular systolic function with an ejection fraction of 30% and a small pericardial effusion (Figure 3). Left heart catheterization demonstrated normal coronary arteries. Right heart catheterization demonstrated minimally elevated filling pressures and borderline cardiac output/index. An endomyocardial biopsy was taken, with pathology showing mild interstitial fibrosis, but no evidence of active myocarditis. Cardiac MRI showed linear mid myocardial LGE in the interventricular septum (Figure 4A) that corresponded with the areas of myocardial edema seen on the T2-weighted images (Figure 4B). There was also elevated native T1 map at 1470 ms (normal 1000 +/− 50 ms at 1.5T) (Figure 4C) along with elevated T2 mapping at 70 ms (myocardial edema suggested if T2 > 55–60 ms) (Figure 4D), and a small pericardial effusion. ECV was unable to be calculated due to an artifact on postcontrast T1 images. This case met the ICOS diagnostic criteria (Table 2, cTn elevation + diagnostic CMR (major criteria)). The patient was started on a pulse dose of corticosteroids for the treatment of presumed myocarditis with significant improvement in symptoms and was discharged home with a stable dose of steroids, as well as goal directed medical therapy for heart failure (beta blockers, ARNI, and SGLT2i). Nivolumab was discontinued. The patient was followed-up in the cardio-oncology clinic and, due to the resolution of symptoms, his steroid treatment was tapered over the next few weeks. During his follow-ups, he showed a complete resolution of clinical symptoms and was able to recover a good exercise tolerance, but unfortunately a repeat echocardiogram at 9 months after the initial presentation showed a persistently reduced ejection fraction.

This case highlights the complexity of the presentation and diagnosis of ICI myocarditis. Despite endomyocardial biopsy being the gold standard, it was not able to establish the diagnosis of myocarditis, likely due to the late presentation and sampling error [62]. The diagnosis was established by cardiac MRI later. As highlighted, multimodal imaging plays an integral part in the diagnosis and management of ICI myocarditis.

## 4. Future Directions

As the research on ICI myocarditis evolves, there is increasing recognition of the clinical heterogeneity of ICI myocarditis and that not all ICI myocarditis cases are the same. There is a subset of patients who do not need aggressive treatment and are able to safely continue ICI treatment. However, some patients will develop severe and potentially fatal ICI myocarditis. It is critically important that future research focuses on differentiating severe ICI myocarditis from the non-severe form using sensitive and specific diagnostic tests (biomarkers or noninvasive imaging or a combination of several tests) in order to render effective and timely treatment to the patients with severe cases while avoiding discontinuing ICIs unnecessarily among the patients with non-severe cases after ICI treatment. Multimodal imaging will play a crucial role in the diagnosis and risk stratification of ICI myocarditis. Data are also lacking on the long-term outcomes of ICI myocarditis, regardless of the severity. There is an evidence gap in the optimal second-line treatment strategy, especially for patients with severe ICI myocarditis. The optimal type of immunosuppressant or targeted therapy and the timing and regimen of combination therapy with corticosteroids are to be studied in future research.

## Figures and Tables

**Table 1:** Prior studies concerning the prevalence of ICI myocarditis in patients treated with immune checkpoint inhibitors.

Study Year and Author	Cohort Year	Cohort Size (# of Patients)	Prevalence of ICI Myocarditis
2016 Johnson et al. [9]	Up to 2016	20,594	0.09%
2018 Mahmood et al. [4]	2013–2017	964	1.14%
2018 Salem et al. [5]	2008–2018	31,321	0.39%
2020 Oren et al. [6]	2010–2019	3326	0.32%
2022 Vasbinder at al. [10]	2014–2021	2606	1.04%
2023 Zadok et al. [11]	2015–2022	10,046	1.2%
2024 Bragheiri et al. [7]	2011–2019	4195	1.8%
2024 Vasbinder et al. [12]	2014–2023	4056	0.8%
2024 Qin et al. [13]	2018–2023	8875	0.35%
2025 Ozaki et al. [14]	2011–2022	88,928	0.48%

**Table 2:** Diagnostic criteria for ICI myocarditis. Adapted from Herrmann et al. [32].

IC-OS 2021 Consensus Criteria for the Diagnosis of ICI-rM		
Pathological diagnosis	Multifocal inflammatory cell infiltrates with overt cardiomyocyte loss on light microscopy of cardiac tissue samples	
Clinical diagnosis	After excluding ACS or acute infectious myocarditis: cTn elevation + 1 major criteria or cTn elevation + 2 minor criteria	Major: diagnostic CMR Minor: clinical syndrome ventricular arrhythmia or conduction system disease decline in cardiac systolic function with/without WMA in a nonTakotsubo pattern other IrAE like myositis, myopathy, or myasthenia gravis suggestive CMR
Modifiers of severity		
Severe	Hemodynamic instability, HF with or without mechanical ventilation, complete or high-grade heart block, ventricular arrhythmia	
Non-severe	Symptomatic but hemodynamically and electrically stable, may have a reduced LVEF, no features of severe disease	
Smoldering	Incidentally diagnosed myocarditis without any clinical signs or symptoms	
Steroid Refractory	Non-resolving or worsening myocarditis (clinical worsening or a persistent troponin elevation after the exclusion of other etiologies) despite high-dose methylprednisolone treatment	
Recovery from Myocarditis		
Complete Recovery	Complete resolution of symptoms, normalization of biomarkers, and recovery of LVEF after the discontinuation of immunosuppression; CMR may show LGE or elevated T1 due to fibrosis, but no edema	
Recovering	Ongoing improvement without normalization in signs, symptoms, biomarkers, and imaging parameters while on tapering doses of immunosuppressants	

The International Cardio-Oncology Society (IC-OS) and European Society of Cardiology published consensus guidelines concerning immune checkpoint inhibitor (ICI) myocarditis, serving as basis for the integration of multimodal imaging into this disease diagnostic pathway. ICIrM = ICI-related myocarditis. cTn = cardiac troponin. WMA = wall motion abnormality. IrAE = immune-related adverse event. Takotsubo = stress cardiomyopathy.

**Table 3:** Guidelines for the management of ICI myocarditis. From Nielson et al. [70].

Time Period or Disease Grade	NCCN Guidelines	SITC Guidelines	ASCO Guidelines	ESMO Guidelines	2022 ESC Guidelines
Baseline	Consider ECG; individualized assessment in consultation with cardiology, as indicated	No recommendation	No recommendation; no clear evidence regarding the efficacy of routine baseline ECG and troponin level measurement; some centers perform baseline testing	Heart rate, blood pressure, ECG, chest radiograph, TTE and levels of troponin I and/or T, CK, NT-pro-BNP; blood electrolytes	Cardiovascular assessment, ECG, TTE (high risk: dual ICI, combination of ICIs and cardiotoxic therapy); non cardiovascular events, prior cardiovascular disease, cancer therapy-related cardiac dysfunction, consider TTE in all patients, cardiac troponin, natriuretic peptides (BNP and NT-proBNP)
Monitoring	Consider periodic testing for patients with abnormal baseline or symptoms	No recommendation	No recommendation; no clear evidence regarding the efficacy of routine serial ECG and troponin level measurements; some centers conduct testing during the initial period of therapy	Repeat before each ICI cycle, measurement of levels of troponin T, NT-pro-BNP, CK, troponin I	ECG, cardiac troponin level measurements in cycles 1,2,and 4 and then every 3 cycles of ICIs; cardiovascular assessment every 3–6 mo for >12 mo: cardiovascular assessment; ECG; measurement of cardiac troponin and natriuretic peptides (BNP and NT-proBNP) levels
Assessment	Alternative reasons for elevation should be ruled out: inflammatory biomarkers, erythrocyte sedimentation rate, levels of C-reactive protein, WBC counts, echocardiography, cardiac MRI; severe symptoms: consider cardiac biopsy	Hospital attendance and consultation with a cardiologist; ECG; measurement of troponin levels, cardiac MRI (with or without right heart catheterization and biopsy)	Early cardiology involvement: ECG, continuous monitoring, measurement of troponin and CK levels; alternative reasons for elevation should be ruled out; echocardiography and cardiac MRI (preferred); guided by cardiology; stress test and catheterization and biopsy if warranted	Clinical ECG biomarker, echocardiography, cardiac MRI (if MRI is not available, cardiac CT or cardiac PET-CT recommended), preferentially with gallium-68-DOTATOC	NA
Grade 1	No recommendation	Suspected ICI-induced myocarditis; methylprednisolone 1000 mg/d, for 3–5 d as soon as possible when diagnosis is likely; permanent discontinuation of ICI should be considered.; admit to coronary care unit; if no response within 24 h consider ATG, MMF, abatacept, alemtuzumab; caution is advised against infliximab; consider transient pacemaker.	Hold ICI if the troponin level is above the upper limit of normal; control troponin level 6 h later; resuming ICI if troponin level normalizes may be considered	Discontinue ICI; first line methylprednisolone, 500–1000 mg for 3 days or until clinically stable; second line MMF or tocilizumab; third line ATG, alemtuzumab, or abatacept	NA
Grade 2	No recommendation		Discontinue ICI within 24 h; initiate prednisone 1–2 mg/kg/d; admit for cardiology consultation; consider immediate transfer to coronary care unit for patients with troponin or conduction abnormalities; consider pacemaker for new conduction delay; for patients with a high clinical suspicion, treatment should be offered empirically; in the absence of immediate response to high dose corticosteroids, administer methylprednisolone, 1000 mg/d, and add MMF, ATG, or infliximab (contraindicated I patients with moderate to severe HF); consider abatacept or alemtuzumab		NA
Grades 3 and 4	Discontinue ICI; consider methylprednisolone, 1000 mg/d, for 3–5 d; without improvement within 24 h, consider transient pacemaker for arrhythmia; consider adding ATG, infliximab (contraindicated in patient with moderate to severe HF), IVIG, MMF; intensive care unit-level monitoring: may be used: alemtuzumab, abatacept				NA

Abbreviations: ASCO, American Society of Clinical Oncology; ATG, antithymocyte globulin; BNP, brain natriuretic peptide; CK, creatinine kinase; CK-MB, CK-myocardial band; ECG, electrocardiogram; ESC, European Society of Cardiology; ESMO, European Society for Medical Oncology; HF, heart failure; ICI, immune checkpoint inhibitor; IVIG, intravenous immunoglobulin; MMF, mycophenolate mofetil; MRI, magnetic resonance imaging; NA, not applicable; NCCN, National Comprehensive Cancer Network; NT-pro-BNP, N terminal pro-B type natriuretic peptide; PET-CT, positron emission tomography and computer tomography; SITC, Society for Immunotherapy of Cancer; TTE, transthoracic echocardiography. Various societal guidelines detailing the management of ICI myocarditis based on clinical acuity. Severity is graded 1–4. Grade 1 = asymptomatic patient with abnormal cardiac biomarkers or EKG. Grade 2 = abnormal screening tests with mild symptoms. Grade 3 = reduced ejection fraction or CMR suggestive of myocarditis. Grade 4 = life-threatening decompensation.
